# Letrozole protects against cadmium-induced inhibition of spermatogenesis via LHCGR and Hsd3b6 to activate testosterone synthesis in mice

**DOI:** 10.1186/s12958-022-00915-4

**Published:** 2022-03-02

**Authors:** Yao Yao, Yangyang Wan, Xiaoyun Shi, Lan Guo, Hui Jiang, Xiansheng Zhang, Bo Xu, Juan Hua

**Affiliations:** 1 Department of Biochemistry and Molecular Biology, School of Basic Medical Sciences, Anhui Medical University, Hefei, 230032 China; 2 Reproductive and Genetic Hospital, The First Affiliated Hospital of USTC, Division of Life Sciences and Medicine, University of Science and Technology of China, Hefei City, Anhui Province China; 3 The Department of Urology, Peking University Third Hospital, Andrology, Peking, 100191 China; 4 Department of Urology, The First Affiliated Hospital of Anhui Medical University, Hefei, China

**Keywords:** Cadmium, Etrozole, Testosterone synthesis, Male infertility, RNA-Seq

## Abstract

The heavy metal cadmium is proposed to be one of the environmental endocrine disruptors of spermatogenesis. Cadmium-induced inhibition of spermatogenesis is associated with a hormone secretion disorder. Letrozole is an aromatase inhibitor that increases peripheral androgen levels and stimulates spermatogenesis. However, the potential protective effects of letrozole on cadmium-induced reproductive toxicity remain to be elucidated. In this study, male mice were administered CdCl_2_ (4 mg/kg BW) orally by gavage alone or in combination with letrozole (0.25 mg/kg BW) for 30 days. Cd exposure caused a significant decreases in body weight, sperm count, motility, vitality, and plasma testosterone levels. Histopathological changes revealed extensive vacuolization and decreased spermatozoa in the lumen. However, in the Cd + letrozole group, letrozole treatment compensated for deficits in sperm parameters (count, motility, and vitality) induced by Cd. Letrozole treatment significantly increased serum testosterone levels, which were reduced by Cd. Histopathological studies revealed a systematic array of all germ cells, a preserved basement membrane and relatively less vacuolization. For a mechanistic examination, RNA-seq was used to profile alterations in gene expression in response to letrozole. Compared with that in the Cd-treated group, RNA-Seq analysis showed that 214 genes were differentially expressed in the presence of letrozole. Gene ontology (GO) enrichment analysis and KEGG signaling pathway analysis showed that steroid biosynthetic processes were the processes most affected by letrozole treatment. Furthermore, we found that the expression of the testosterone synthesis-related genes LHCGR (luteinizing hormone/choriogonadotropin receptor) and Hsd3b6 (3 beta- and steroid delta-isomerase 6) was significantly downregulated in Cd‐treated testes, but these genes maintained similar expression levels in letrozole-treated testes as those in the control group. However, the transcription levels of inflammatory cytokines, such as IL-1β and IL-6, and oxidative stress-related genes (Nrf2, Nqo1, and Ho-1) showed no changes. The present study suggests that the potential protective effect of letrozole on Cd-induced reproductive toxicity might be mediated by the upregulation of LHCGR and Hsd3b6, which would beneficially increase testosterone synthesis to achieve optimum protection of sperm quality and spermatogenesis.

**Supplementary Information:**

The online version contains supplementary material available at 10.1186/s12958-022-00915-4.

## Introduction

The incidence of decreased fertility is a public health problem because of its high prevalence and its serious social impact of couples globally are infertile, and half of these incidences of infertility are due to male infertility [1]. The heavy metal cadmium (Cd) is postulated to be one of the environmental endocrine disruptors causing male infertility [2]. Several studies have revealed that Cd can induce severe testicular toxicity through a series of complications: reducing testicular weight; inducing testicular hemorrhage; and reducing sperm cell count, sperm motility, and testosterone hormone concentrations [3, 4]. Disturbed hormonal production is presumed to play a major role in the pathogenesis of infertility and testicular dysfunction induced by cadmium [5]. According to some studies, Cd significantly decreases the serum testosterone (T) level by inhibiting the activities of steroidogenic enzymes [3, 6]. Several mechanisms of cadmium-induced disruptions in hormone production have been proposed. The first suggests that Cd can directly binds to estrogen receptors and androgen receptors [7]. In the second mechanism, Cd alters the expression of enzymes related to steroidogenesis, such as StAR, cholesterol C20-22 desmolase, 17α-hydroxylase, and 17β-hydroxysteroid dehydrogenase, and suppresses the expression of the LH receptor [8]. However, the mechanisms underlying this anti-steroidogenic effect remain largely undiscovered. Considering the severity of cadmium contamination and its testicular toxicity, the identification of therapeutic or preventive interventions for cadmium-induced male infertility is urgently needed [9]. Since cadmium exerts its deleterious effects on the testis by disturbing hormone production [3], aromatase inhibitors can potentially prevent cadmium-induced testicular dysfunction.

Letrozole is a reversible type 2 aromatase inhibitor that binds to cytochrome P-450 and inhibits the conversion of testosterone to estradiol and androstenedione to estrone [10]. Therefore, it increases the levels of testosterone and stimulates spermatogenesis [11]. A few scientific studies also seem to support the therapeutic potential of letrozole in male reproductive health [12]. Letrozole has been reported to effectively improve sperm parameters in infertile men with low serum testosterone/estradiol levels and increase the chance of successful conception in couples including men with idiopathic severe oligozoospermia [13–15]. However, the potential use of letrozole could be used as a protectant against exposure to harmful reproductive toxicants remains unclear. Therefore, there is a need for a mechanistic approach is needed to validate the efficacy of letrozole as an aphrodisiac treatment that protects the male reproductive organs from toxic chemicals and serve as a medicine for male infertility treatment.

Therefore, the present study aims to investigate the potential protective effect of letrozole on CdCl_2_-induced testicular toxicity in male mice. In addition, the possible mechanisms underlying this effect are clarified.

## Materials and methods

### Animals and experimental design

Five-week-old male ICR mice were purchased from Anhui Medical Laboratory Animal Center (Hefei, China) and acclimated for one week before the experiments. All mice were housed in a room with constant temperature (22–24 °C) and a 12/12 h light–dark cycle, and they were allowed access to food and water ad libitum. In preliminary experiments, the mice were randomly divided into 3 groups (*n* = 3 mice per group): the control group, the low Cd-treated group using 2.5 mg/kg/day cadmium chloride (Sigma-Aldrich, USA), and the high Cd-treated group (4 mg/kg/day). Subsequently, the experiments were conducted with a high cadmium dose that was co-administered along with three different letrozole (Jiangsu Hengrui Medicine Co.,Ltd, China) concentrations (0.25, 0.3, or 0.35 mg/kg/day letrozole) to determine the optimal concentration. In formal experiments, the animals were randomly divided into 3 groups (*n* = 8 mice per group): the control group, the Cd-treated group (4 mg/kg/day cadmium chloride dissolved in distilled water), and the letrozole plus Cd group (0.25 mg/kg/day letrozole plus cadmium chloride). The control group received only an equal volume of distilled water. The mice were euthanized, and body weights were recorded after 30 days.

### Ethical compliance

This work was consented by the ethics committee of Anhui Medical University (Approve ID:20,200,054).

### Epididymal sperm analysis and testes weight

The left cauda epididymis was placed in 200 µl of DMEM (Gibco, USA) at 37 °C, cut into small pieces and and sperm were released by incubating the tissue fragments at 37 °C for 3 min. The sperm suspension was placed in the sperm counting plate and counted by using a method described as previously reported [16]. Also the sperm suspension was analyzed by a computer-assisted semen analysis (CASA, Song Jing Tian Lun Biotechnology Co., Ltd., Nanning, China) system for sperm motility and vitality according to the manufacturer’s protocol as previously described [17, 18].

The testes on both sides were removed and weighed. The gonadosomatic index was calculated using the formula [testicular weight (g)/body weight (g)] × 100%.

### Histological analysis

The left testes of mice were placed in 4% paraformaldehyde (PFA) for paraffin embedding. The paraffin-embedded tissues were sectioned into 5 µm slices and stained with hematoxylin and eosin (H&E). Testicular sections from three mice in each group were randomly selected to count abnormal seminiferous tubules after HE staining and used to calculate the abnormal rate of seminiferous tubules.

### RNA extraction and quantitative real-time PCR

Total RNA was extracted from the testes using TRIzol reagent (Invitrogen, USA) according to the manufacturer’s protocol and reverse transcribed into complementary DNA (cDNA) templates using a cDNA reverse transcription kit (Novoprotein, China). Quantitative real-time PCR was performed using SYBR qPCR SuperMix Plus (Novoprotein, China). The amplification of cDNA templates was performed using a real-time fluorescent quantitative PCR detection sysQtem (Roche) with the following procedure: denaturation at 95 ℃ for 1 min followed by 40 cycles at 95 ℃ for 20 s, 60 ℃ for 1 min, and 95 ℃ for 10 s, 65 ℃ for 60 s, 97 ℃ for 1 s, 37 ℃ for 30 s. The internal reference gene was β-actin.

### Serum hormone analysis

The serum hormone level was analyzed as previously described [2]. The collected blood was incubated at room temperature for 1 h and centrifuged at 1500 g for 10 min at 4 ℃ to obtain serum. Serum concentrations of luteinizing hormone, estrogen and testosterone in serum were determined using enzyme-linked immunosorbent assay (ELISA) kit (Lanso, China).

### Transcriptome sequencing and analysis

The right testes of mice (*n* = 3 animals per group) were removed and quickly placed on dry ice. Total RNA was extracted using the mirVana miRNA Isolation Kit (Ambion) according to the manufacturer’s protocol. RNA integrity was evaluated using an Agilent 2100 Bioanalyzer (Agilent Technologies, Santa Clara, CA, USA). The libraries were constructed using the TruSeq Stranded mRNA LT Sample Prep Kit (Illumina, San Diego, CA, USA) according to the manufacturer’s instructions. Then, these libraries were sequenced on the Illumina sequencing platform (HiSeqTM 2500 or Illumina HiSeq X Ten), and 125 bp/150 bp paired-end reads were generated.

The FPKM and read count values of each transcript were calculated using bowtie2 and eXpress. DEGs were identified using the DESeq functions: estimateSizeFactors and nbinomTest. A *P* value < 0.05 and fold Change > 1.5 (or fold Change < 0.67) was set as the threshold for significantly differential expression. A hierarchical clustering analysis of DEGs was performed to explore transcript expression patterns. GO enrichment and KEGG pathway enrichment analyses of DEGs were performed using R software based on the hypergeometric distribution.

### Western blot

Tissue lysates were prepared with RIPA buffer plus phenylmethane sulfonyl fluoride (PMSF) and protease inhibitors before the experiments. Proteins were extracted from testes and loaded onto 10% SDS–polyacrylamide gel for electrophoresis and the isolated proteins were transferred to the NC membrane. The samples were analyzed by Western blot using antibodies for LHCGR, Cyp11a1, Cyp17a1 (ABclonal, China) and β-actin (Affinity, USA).

### Statistical analysis

GraphPad Prism 8.0 software was used for graphical presentation and data analysis. All data are presented as the means ± standard errors (SEM). The qPCR data used to validate transcriptome sequencing results were analyzed using an unpaired t test. The other data were analyzed using one-way analysis of variance (ANOVA) followed by Bonferroni's multiple comparison test as a post hoc comparison. *P* < 0.05 was consider to be statistically significant.

## Results

### Effects of letrozole on body weight and testes coefficients in cadmium-exposed mice

The body weight of animals treated with cadmium alone significantly decreased compared to that of the control group (Table 1). Furthermore, the administration of letrozole and CdCl_2_ significantly increased body weight compared to the Cd group (Table 1). No significant differences in the absolute and relative testis weights were observed between animals treated with cadmium alone or with cadmium followed by letrozole and control animals (Table 1).

**Table 1:** Effects of CdCl_2_ alone and in combination with letrozole (CdCl_2_ + letrozole) on the body weight and absolute and relative testes weights of male mice

Groups	Body weight		TW	TW/BW ratio (mg/g)
	Initial BW	Final BW		
Control	30.52 ± 0.41	35.97 ± 0.57	0.25 ± 0.01	0.68 ± 0.02
Cd (4 mg/kg)	30.99 ± 0.64	33.55 ± 0.53*	0.24 ± 0.01	0.73 ± 0.03
Cd + letrozole (4 + 0.25 mg/kg)	30.85 ± 0.21	34.89 ± 0.31^#^	0.25 ± 0.01	0.72 ± 0.03

BW Body weight, TW Testis weight. Means ± SEM of five animals in each group. Compared with control group, *p < 0.05; compared with the Cd group, #*p* < 0.05

### Effects of letrozole on sperm functional parameters and testicular histopathology in cadmium-exposed mice

**Fig. 1:**
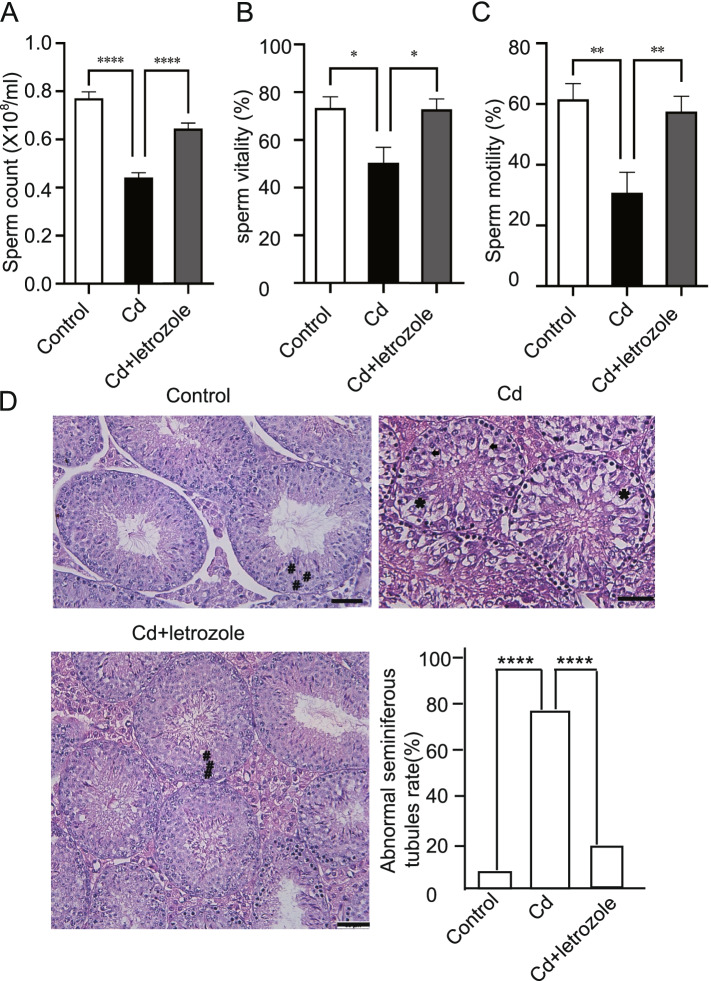
Effect of letrozole on sperm characteristics and testicular histopathology in CdCl_2_-treated male mice. **A** Sperm count (sperm/ml). **B** Sperm motility. **C** Sperm viability. **D** Testicular histopathology (H&E staining) of mice treated with Cd and letrozole (germ cells loss (asterix) intercellular vacuolization (arrow); germ cell (#); Scale = 50 *um*)

Compared with the control group, sperm count (*p* < 0.0001), sperm vitality (*p* = 0.021), and motility (*p* = 0.0047) were significantly decreased in animals treated with cadmium alone. On the other hand, in animals treated with cadmium followed by letrozole, sperm count (*p* < 0.0001), sperm vitality (*p* = 0.0119) and motility (*P* = 0.0055) increased compared to cadmium- treated animals, and no statistical significance was observed in sperm vitality (*p* = 0.996) and sperm motility (*p* = 0.83) of Cd-letrozole treated group compared to control (Fig. 1A-C). Upon a histological examination of the testis structure, no changes were observed in the control testes. In contrast, many marked histopathological alterations were noticed in the testes of the CdCl_2_-treated group, with a significant increase in the number of affected seminiferous tubules (Fig. 1D). Some seminiferous tubules were lined by Sertoli cells and a few germ cells or by a single layer of germ cells (Fig. 1D). The findings from the CdCl_2_-and letrozole-treated groups revealed that letrozole could partially restored spermatogenesis, as evidenced by a gradual increase in the number of germ cell layers, with a decrease in the percentage of affected seminiferous tubules (Fig. 1D).

### Effects of letrozole on serum levels of LH, testosterone and testicular mRNA levels of caspase-3 and bcl-2 in cadmium-exposed mice

**Fig. 2:**
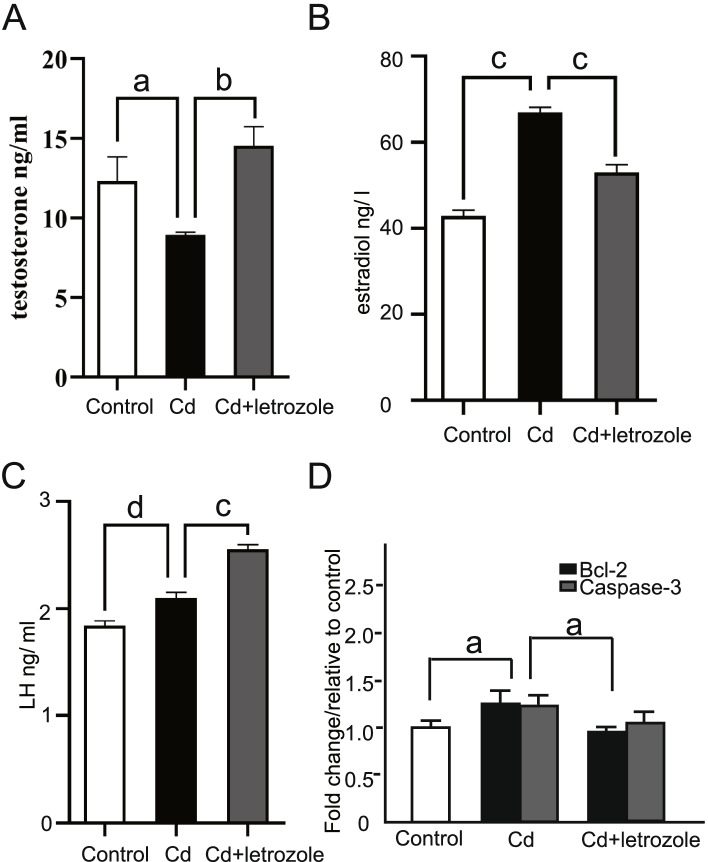
Effects of letrozole on CdCl_2_-induced changes in serum testosterone levels, estradiol levels, LH levels, and caspase-3 and Bcl-2 mRNA expression levels. **A** Serum testosterone level. **B** Estradiol level. **C** LH level. **D** Caspase-3 and Bcl-2 mRNA levels in the testes. Data are presented as the means ± SEM of 6 mice per group. a: > 0.05, b: 0.01 < *p* < 0.05, c: *p* < 0.0001, d:0.001 < *p* < 0.005

The serum testosterone levels of animals supplemented with letrozole after cadmium exposure were estimated after 30 days of oral gavage. As presented in Fig. 2A, cadmium exposure significantly decreased the serum testosterone level compared to the control group. On the other hand, significantly higher serum testosterone levels were detected in the CdCl_2_-and letrozole-treated groups. According to a previous study, letrozole increases the LH level by reducing the estrogen level while also increasing the testosterone level [19]. Therefore, we detected the levels of E_2_ and LH in the CdCl_2_- and Cd-letrozole treated groups. As shown in Fig. 2B, C, letrozole decreased the serum estrogen concentration and increased the LH level compared with the Cd group. Next, we investigated the effects of letrozole on the levels of the Bcl-2 and caspase-3 transcripts, and no significant differences were observed in animals treated with cadmium alone or with cadmium followed by letrozole compared with control animals (Fig. 2D).

### Effects of letrozole on transcription in the testis of cadmium-exposed mice

**Fig. 3:**
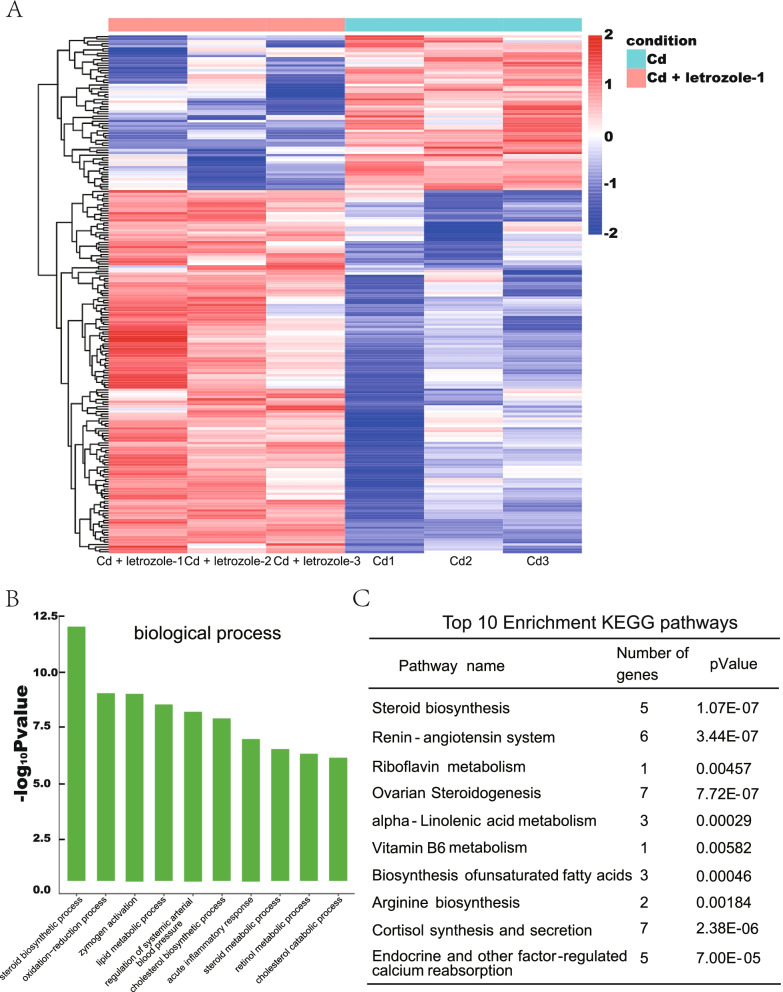
Analysis of differentially expressed genes in letrozole-treated mice. **A** Hierarchical clustering analysis of gene expression profiles. Each column represents one mouse, and each horizontal line refers to a gene. The color legend is shown at the top-left of the figure. Red indicates genes with higher expression relative to the geometrical means; blue indicates genes with lower expression relative to the geometrical means. **B** Biological process Gene Ontology (GO) analysis of the biological processes in which differentially expressed genes were enriched. **C** Top 10 enriched KEGG pathways

**Fig. 4:**
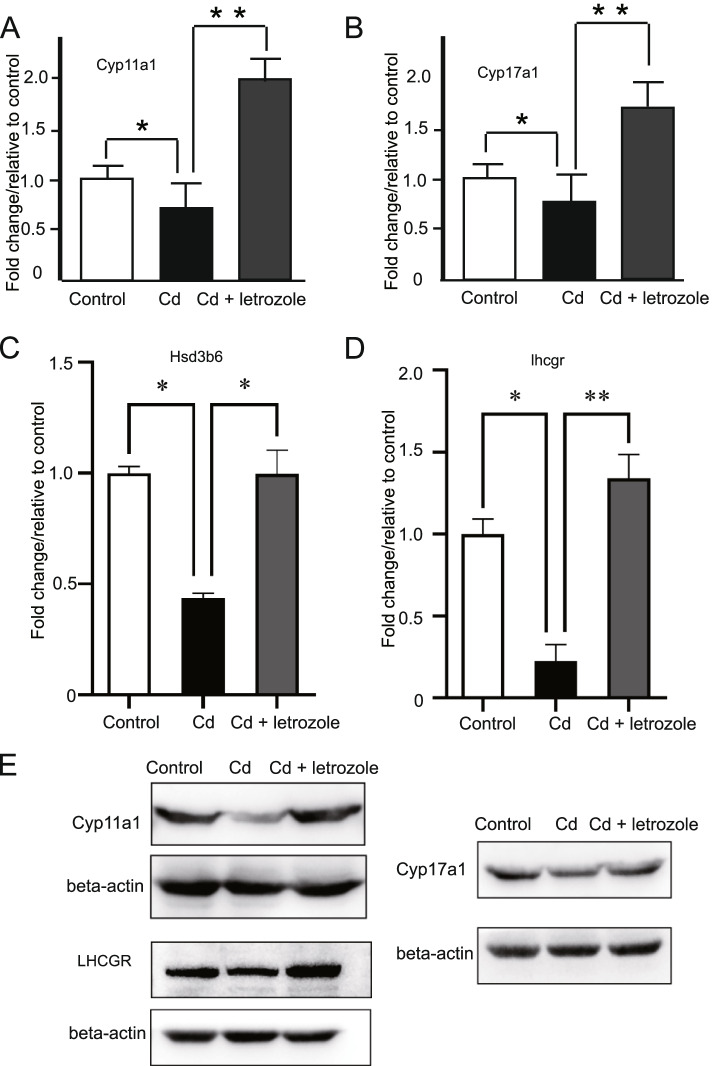
Effects of CdCl_2_ alone and in combination with letrozole (CdCl_2_ + letrozole) on the expression of Cyp11a1, Cyp17a1, Hsd3b6 and LHCGR in mouse testes. **A** The mRNA expression level of Cyp11a1. **B** The mRNA expression level of Cyp17a1. **C** The mRNA expression level of Hsd3b6. **D** The mRNA expression level of LHCGR. E. the protein expression level of Cyp11a1, Cyp17a1 and LHCGR in mouse testes treated with Cd or Cd + letrozole. *p < 0.05, **p < 0.01. The means ± SEM were calculated for the 6 mice in each group

RNA-seq was used to examine changes in the testis transcriptome in response to Cd and Cd + letrozole. Compared to that in the Cd-treated group, the RNA-Seq analysis showed that 214 genes were differentially expressed in animals treated with letrozole (Fig. 3A). First, we performed a qPCR analysis of six genes to validate the RNA-Seq data (Supplementary Fig. 1), the qPCR results showed that these genes exhibited similar expression levels to those detected using RNA-seq. Next, differentially expressed genes were functionally classified by performing a GO enrichment analysis to obtain a comprehensive understanding of the effect of letrozole on testicular gene expression. These genes were classified into several GO categories according to their functions in various biological processes. The GO enrichment analysis showed that representative genes participating in steroid biosynthetic processes, oxidation–reduction processes, and acute inflammatory responses were significantly differentially expressed in response to letrozole (Fig. 3B), and the most enriched GO category contained the genes associated with steroid biosynthetic processes. The steroid biosynthetic process-related categories contained 11 genes; ten genes were upregulated, and only Cyp21a1 was downregulated in the letrozole group. Among these genes, half were specifically responsible for testosterone synthesis, suggesting that testosterone synthesis occurred in response to letrozole treatment. In addition, the top 5 KEGG signaling pathways affected by letrozole treatment were steroid biosynthesis, the renin-angiotensin system, riboflavin metabolism, ovarian steroidogenesis, and α-linolenic acid metabolism (Fig. 3C). Among these represented pathways, steroid biosynthesis was also the most enriched among the identified pathways. We determined whether Cd exerted an effect on the expression of these steroid biosynthesis process-related genes by measuring the expression of the Cyp17a1, Cyp21a1, Hsd3b6, Hsd3b7, Hsd17b7, and Cyp11a1 mRNAs in the testes of mice treated with Cd using qPCR. As shown in Fig. 4A-C, the testicular Cyp11a1, Cyp17a1, and Hsd3b6 mRNA levels were significantly decreased in Cd-treated mice. However, the expression of Hsd3b7 and Hsd17b7 was not significantly different in Cd-treated mice (Supplementary Fig. 2). Cyp11a1, Cyp17a1 and Hsd3b6 are involved in the conversion of cholesterol to testosterone in Leydig cells, and the upstream regions of these genes are LHCGR and LH. We also found that letrozole significantly increased the expression of LHCGR and that Cd decreased the expression level of LHCGR (Fig. 4D). In addition, significantly increased LH levels were observed in the Cd + letrozole group, indicating that letrozole activated testosterone synthesis via the LHCGR-Hsd3b6 pathway (Fig. 2C). Western blotting results supported the results of the expression level of mRNA and showed decreased protein expression levels of Cyp11a1, Cyp17a1, and LHCGR in testes of Cd-treated group compared with normal control. Moreover, Cd + letrozole treated group showed significantly increased Cyp11a1, Cyp17a1, and LHCGR expression levels compared with Cd alone–treated group (Fig. 4E).

## Discussion

Cadmium, a common environmentally toxic heavy metal, is widely used in various applications and is present in almost every location in the environment [5]. To date, an increasing number of studies have shown that exposure to cadmium causes severe testicular injury and subsequent infertility in experimental animals [5]. The human population is exposed to cadmium mostly through food, water, cigarette smoke, and industrial or agricultural products [2]. Therefore, an increasing number of people are focusing on the toxicological effects of cadmium on male infertility. In recent years, several researchers have used different approaches to mitigate cadmium-induced testicular toxicity. As oxidative stress and inflammation are important contributors to cadmium-mediated testicular damage, some products with antioxidant and anti-inflammatory properties have been used to reduce the testicular toxicity of Cd, such as curcumin, grape seed extract, coenzyme Q10, green tea extract, alpha-tocopherol, melatonin, vitamin E, and selenium and Fragaria × ananassa crude extract [20–26]. Additionally, Martin et al. reported that FK506, a calcineurin inhibitor, prevents cadmium-induced testicular toxicity in mice [27]. Disturbed gonadal and hormonal functions are also postulated to play a crucial role in the testicular toxicity induced by cadmium. Previous studies have described that several substrates that can regulate steroidogenesis and exert therapeutic effects on cadmium-induced testicular toxicity, such as Feijoa, Shilajit and Moringa leaf ethanolic extracts [6, 28, 29]. The present study was conducted to evaluate the effect of cadmium on steroidogenesis, the quality and quantity of sperm, and the inflammatory response in mice. The results showed that Cd exposure reduced body weight, significantly decreased the sperm count, sperm motility, sperm viability, and serum testosterone concentrations in mice. Moreover, a histological examination of the testis structure showed an abnormal seminiferous tubule structure and decreased Leydig cell numbers. Treatment with letrozole restored the weights of reproductive organs affected by cadmium. Additionally, the administration of letrozole ameliorated the CdCl_2_-induced changes in histology, sperm characteristics and serum testosterone levels. Based on these results, CdCl_2_ toxicity induced serious alterations in the testes, which were prevented by the coadministration of letrozole.

The potentially protective mechanism of letrozole on cadmium-induced testicular toxicity in mice was discovered by use RNA-seq to analyze the transcriptome of the mouse testes after letrozole treatment. The bioinformatics analysis revealed that the expression of many genes was modulated by letrozole. Ontology enrichment analysis provided a noteworthy focus on steroid biosynthetic processes. We observed an increase in the expression of Cyp11a1, Cyp17a1, Ren1, and Retsat in the letrozole group, which was reported to be downregulated after cadmium exposure in a previous studies [30, 31]. Additionally, significant increases in Cyp21a1 Hsd3b6, Hsd3b7, and Hsd17b7 expression were observed in the letrozole group. As these genes are related to testosterone synthesis, we presumed that letrozole protects against cadmium-induced inhibition of spermatogenesis by inducing testosterone synthesis. A previous systematic review validated that the testicular toxicity of Cd is certainly linked to the inhibition of testosterone synthesis [2]. However, the mechanism of testosterone synthesis disturbed by Cd treatment remains unknown. In the current study, Cd treatment caused significant decreases in testicular mRNA expression levels of Cyp11a1, Cyp17a1 and Hsd3b6 compared to controls. Interestingly, the expression of LHCGR, which is upstream of Cyp11a1, Cyp17a1 and Hsd3b6, was downregulated in the testes of Cd-treated mice, and letrozole upregulated LHCGR expression in the testes. Many studies have shown that the negative effects of cadmium on disturbing hormone functions and activating the inflammatory response and cause oxidative stress [32]. Many researchers have shown that the transcription levels of inflammatory cytokines such as IL-1β and IL-6 are significantly increased in Cd-treated mice compared to control mice [32]. Additionally, significantly lower expression of oxidative stress-related genes such as Nrf2, Nqo1 and Ho-1 was observed in the Cd-treated group than in the control group [33]. However, the RNA-seq and qPCR results showed that letrozole treatment did not alter the expression levels of these genes (Supplementary Fig. 3), suggesting that letrozole treatment might not exert a protective effect on oxidative stress and the inflammatory response caused by Cd in the testes. According to these results, we suggest that letrozole protects against cadmium-induced inhibition of spermatogenesis via LHCGR and Hsd3b6 to stimulate testosterone synthesis. However, further investigations are required to confirm this hypothesis.

In summary, our findings revealed that treatment with letrozole can ameliorates Cd-intoxication-induced testicular injury in mice by restoring the normal histological structure and inducing testosterone synthesis through the LHCGR and Hsd3b6 pathways.

## Supplementary Information

**Additional file 1: Supplementary Figure 1.** qPCR validation of the RNA-seq data.

**Additional file 2: Supplementary Figure 2.** The mRNA expression levels of Hsd3b7 and Hsd17b7 in the Cd treatment group and CdCl2 + letrozole group. ns: Not significant, **p < 0.01.

**Additional file 3: Supplementary Figure 3.** The mRNA expression levels of inflammatory cytokines and oxidative stress-related genes in the CdCl2 + letrozole group. ns: not significant.

## Data Availability

The data generated or analyzed during this study are included in this published article. The datasets of variants for this study can be found in the NCBI SRA database (SUB9892257).

## Abbreviations

ANOVA: Analysis of Variance
Bcl-2: B cell leukemia/lymphoma 2
BW: Body weight
CASA: Computer-assisted semen analysis
Cd: Cadmium
CdCl_2_: Cadmium chloride
cDNA: Complementary Deoxyribonucleic Acid
CO: Combined administration
Cyp11a1: Cytochrome P450, family 11, subfamily a, polypeptide 1
Cyp17a1: Cytochrome P450, family 17, subfamily a, polypeptide 1
Cyp21a1: Cytochrome P450, family 21, subfamily a, polypeptide 1
DEGs: Differentially expressed genes
DMEM: Dulbecco’s modified eagle’s medium
E2: Estradiol
ELISA: Enzyme-linked immunosorbent assay
FK506: Tacrolimus
FPKM: Fragments per Kilobase Million
GO: Gene ontology
HE: Hematoxylin–eosin
Ho-1: Heme oxygenase 1
Hsd17b7: Hydroxysteroid (17-beta) dehydrogenase 7
Hsd3b6: 3 Beta- and steroid delta-isomerase 6
Hsd3b7: 3 Beta- and steroid delta-isomerase 7
ICR: Institute of Cancer Research
IL-1β: Interleukin-1β
IL-6: Interleukin-6
KEGG: Kyoto encyclopedia of genes and genomes
LH: Luteinizing hormone
LHCGR: Luteinizing hormone receptor
Nqo1: NAD(P)H dehydrogenase, quinone 1
Nrf2: Nuclear factor, erythroid 2 like 2
NCBI: National Center for Biotechnology Information
NC: Nitrocellulose
PFA: Paraformaldehyde
PMSF: Phenylmethylsulfonyl fluoride
qPCR: Real-time quantitative polymerase chain reaction
R: The R Programming Language
Ren1: Renin 1 structural
RNA: Ribonucleic Acid
RIPA: Radio Immunoprecipitation Assay
RNA-seq: RNA sequencing
SEM: Standard error
StAR: Steroidogenic acute regulatory protein
SDS: Sodium dodecyl sulfate
T: Testosterone
