# Supplementary figures and images for: Letrozole protects against cadmium-induced inhibition of spermatogenesis via LHCGR and Hsd3b6 to activate testosterone synthesis in mice
Source: Reprod Biol Endocrinol. 2022 Mar 2;20:43. doi: 10.1186/s12958-022-00915-4 (PMC8889770; doi:10.1186/s12958-022-00915-4)

# Supplementary Figure. 1

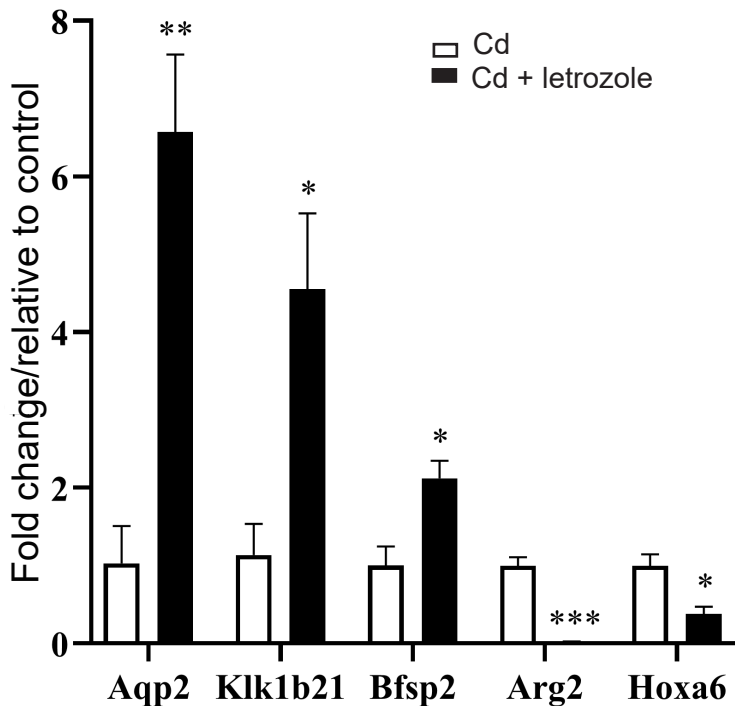

Supplement: Supplementary file 1 — Additional file 1: Supplementary Figure 1. qPCR validation of the RNA-seq data. [file 12958_2022_915_MOESM1_ESM.pdf]

# Supplementary Figure. 2

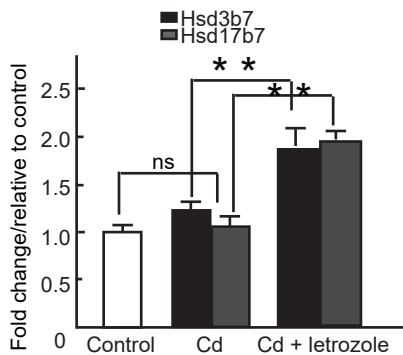

Supplement: Supplementary file 2 — Additional file 2: Supplementary Figure 2. The mRNA expression levels of Hsd3b7 and Hsd17b7 in the Cd treatment group and CdCl2 + letrozole group. ns: Not significant, **p < 0.01. [file 12958_2022_915_MOESM2_ESM.pdf]

# Supplementary Figure. 3

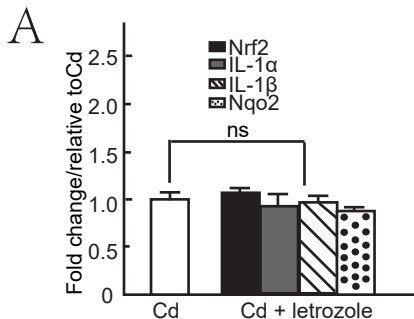

Supplement: Supplementary file 3 — Additional file 3: Supplementary Figure 3. The mRNA expression levels of inflammatory cytokines and oxidative stress-related genes in the CdCl2 + letrozole group. ns: not significant. [file 12958_2022_915_MOESM3_ESM.pdf]
